# Characterization of the complete chloroplast genome of *Suaeda salsa* (Amaranthaceae/Chenopodiaceae), an annual succulent halophyte

**DOI:** 10.1080/23802359.2019.1623113

**Published:** 2019-07-10

**Authors:** Xiao-Jian Qu, Xiao-Tong Li, Luo-Yan Zhang, Xue-Jie Zhang, Shou-Jin Fan

**Affiliations:** Key Lab of Plant Stress Research, College of Life Sciences, Shandong Normal University, Ji’nan, China

**Keywords:** *Suaeda salsa*, plastome, phylogenomics

## Abstract

*Suaeda salsa*, an annual succulent halophytic herb, is one of the major halophyte widely distributed in both saline inland and the intertidal zone. In this study, we report the complete chloroplast genome (plastome) of *S. salsa*. The plastome was 151,642 bp in length and comprises a large single-copy region (83,502 bp), a small single-copy region (17,780 bp), and a pair of inverted repeats (25,180 bp). It encodes 113 unique genes, including 79 protein-coding genes (PCGs), 30 tRNAs, and four rRNAs. The overall GC content of this plastome was 36.4%. Phylogenomic analysis based on 20 plastomes revealed that *S. salsa* was closely related to *S. malacosperma*.

*Suaeda salsa* (Amaranthaceae/Chenopodiaceae) is an annual leaf-succulent halophytic herb with tolerance to salt (Chen et al. 2010; Song and Wang 2015). This species is distributed in Europe and Asia, and it is one of the major halophyte widely distributed in both saline inland and the intertidal zone of northern China (Sui et al. 2010; Song et al. 2016). *Suaeda salsa* grows better in littoral saline soils than in saline inland soils of arid zones (Li et al. 2012; Liu et al. 2018), and it has high salt tolerance during seed germination and seedling stage (Zhou et al. 2016; Song et al. 2017). With no salt glands or bladders (Yang et al. 2010), *S. salsa* is adapted to saline soils through its ability to hyper-accumulate Na^+^ and Cl^−^ in its succulent leaves (Guo et al. 2015) and is capable of removing salts and heavy metals from saline soils (Wang et al. 2015). Furthermore, *S. salsa* roots of the intertidal population could accumulate more Na^+^ and Cl^−^ in both the cortex and the stele than that of the inland population (Song et al. 2011). In addition, salinity can improve chilling resistance and reproductive capacity of *S. salsa* (Cheng et al. 2014; Guo et al. 2018). *Suaeda salsa* is valuable and has been applied as a model halophyte for understanding salt tolerance (Chen et al. 2016). In this study, we reported the plastome of *S. salsa*, which would provide fundamental genetic resource for studying this important species.

Fresh leaves of *S. salsa* were collected from Kenli District (Shandong, China; 37°42′N, 118°58′E). Voucher specimen (hgwz-1) was deposited at College of Life Sciences, Shandong Normal University. Total genomic DNA was extracted by the modified CTAB method described in Wang et al. (2013). Due to limited fresh sample, the plastid DNA was not directly extracted (Liu et al. 2017). The total genomic DNA was used for library preparation and paired-end (PE) sequencing by the Illumina MiSeq instrument at Novogene (Beijing, China). The plastome was assembled using Organelle Genome Assembler (OGA, https://github.com/quxiaojian/OGA) described in Qu (2019). Annotation was performed with Plastid Genome Annotator (PGA, https://github.com/quxiaojian/PGA) (Qu et al., 2019), coupled with manual correction using Geneious v9.1.4 (Kearse et al. 2012). To determine the phylogenetic placement of *S. salsa*, a maximum-likelihood (ML) tree was reconstructed using RAxML v8.2.10 (Stamatakis 2014), including tree robustness assessment using 1000 rapid bootstrap replicates with the GTRGAMMA substitution model, based on the alignment of 79 shared PCGs using MAFFT v7.313 (Katoh and Standley 2013).

The complete plastome of *S. salsa* (GenBank accession number: MK867772) was 151,642 bp in length and comprises a large single-copy region (LSC: 83,502 bp), a small single-copy region (SSC: 17,780 bp), and a pair of inverted repeats (IR: 25,180 bp). This plastome encodes 113 unique genes, including 79 protein-coding genes (PCGs), 30 tRNAs, and four rRNAs. The overall GC content was 36.4%. A total of 113 unique genes were annotated in this plastome, including 79 protein-coding genes (PCGs), 30 tRNAs, and four rRNAs. Among them, eleven PCGs and six tRNAs contained introns, in which nine PCGs and six tRNAs contained one intron and two PCGs contained two introns. There were 18 duplicated genes in the IR. The ML phylogenetic tree showed that *S. salsa* was closely related to *S. malacosperma* (Figure 1).

**Figure 1.**
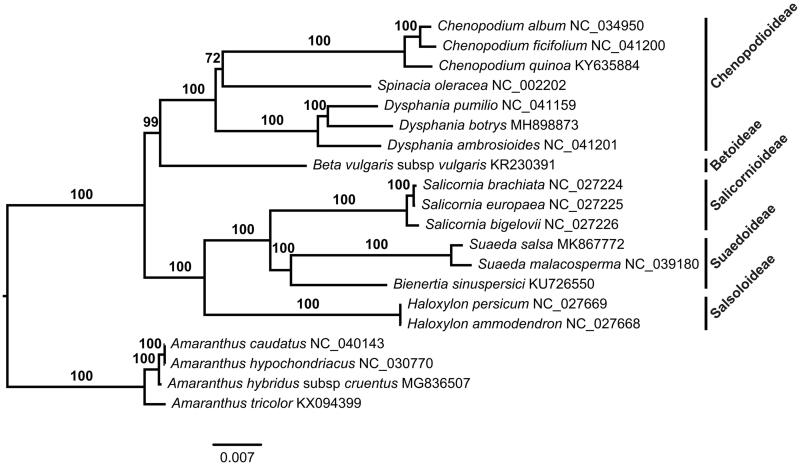
A maximum likelihood (ML) tree inferred from 79 plastome genes is shown. Four *Amaranthus* species from Amaranthaceae are used as outgroup. The numbers on branches are bootstrap support values.
